# Towards Efficient Electricity Forecasting in Residential and Commercial Buildings: A Novel Hybrid CNN with a LSTM-AE based Framework

**DOI:** 10.3390/s20051399

**Published:** 2020-03-04

**Authors:** Zulfiqar Ahmad Khan, Tanveer Hussain, Amin Ullah, Seungmin Rho, Miyoung Lee, Sung Wook Baik

**Affiliations:** Intelligent Media Laboratory, Digital Contents Research Institute, Sejong University, Seoul 143-747, Korea; mzulfiqar3797@gmail.com (Z.A.K.); tanveerkhattak3797@gmail.com (T.H.); qamin3797@gmail.com (A.U.); smrho@sejong.ac.kr (S.R.); miylee@sejong.ac.kr (M.L.)

**Keywords:** buildings energy management, deep learning, energy consumption prediction, LSTM, autoencoder, load forecasting, smart sensors

## Abstract

Due to industrialization and the rising demand for energy, global energy consumption has been rapidly increasing. Recent studies show that the biggest portion of energy is consumed in residential buildings, i.e., in European Union countries up to 40% of the total energy is consumed by households. Most residential buildings and industrial zones are equipped with smart sensors such as metering electric sensors, that are inadequately utilized for better energy management. In this paper, we develop a hybrid convolutional neural network (CNN) with an long short-term memory autoencoder (LSTM-AE) model for future energy prediction in residential and commercial buildings. The central focus of this research work is to utilize the smart meters’ data for energy forecasting in order to enable appropriate energy management in buildings. We performed extensive research using several deep learning-based forecasting models and proposed an optimal hybrid CNN with the LSTM-AE model. To the best of our knowledge, we are the first to incorporate the aforementioned models under the umbrella of a unified framework with some utility preprocessing. Initially, the CNN model extracts features from the input data, which are then fed to the LSTM-encoder to generate encoded sequences. The encoded sequences are decoded by another following LSTM-decoder to advance it to the final dense layer for energy prediction. The experimental results using different evaluation metrics show that the proposed hybrid model works well. Also, it records the smallest value for mean square error (MSE), mean absolute error (MAE), root mean square error (RMSE) and mean absolute percentage error (MAPE) when compared to other state-of-the-art forecasting methods over the UCI residential building dataset. Furthermore, we conducted experiments on Korean commercial building data and the results indicate that our proposed hybrid model is a worthy contribution to energy forecasting.

## 1. Introduction

Electrical energy consumption has recently been accelerating due to rapid population and economic growth [1]. According to the World Energy Outlook (2017), global energy demand is predicted to increase by 1.0% compound annual growth rate (CAGR) over the period of 2016‒40 [2]. Residential buildings play a vital role in this consumption, constituting 27% of total global energy usage, and have a substantial impact on overall energy consumption [3]. In the US, buildings make up 40% of their national overall energy usage [4]. Due to the high level of electricity consumption in commercial and residential buildings, efficient smart electrical energy prediction and its management are becoming more important because the load forecasting directly affects the control and planning of power systems’ operation. A research study estimated that a 1% decrease in forecasting errors can save £10 million per year for the UK power system [5]. Therefore, appropriate energy planning plays a vital role in saving energy, as well as being an economical solution. Future energy planning is possible through computationally intelligent electricity forecasting methods [6,7].

Electricity consumption prediction is a multivariate time series problem where the sensors generate data that may contain uncertainty [8,9], redundancy, missing values, etc. Due to irregular trend components and seasonal patterns, it is difficult to accurately predict electricity consumption by employing traditional machine learning models [10]. On the other hand, deep learning models yield ultimately better results and are less error prone. Deep learning models are aggressively studied in several applications such as CNNs, which are superior at recognizing images, and recurrent neural networks (RNNs) [11], which perform well in natural language processing (NLP) [12] and speech recognition problems. In recent studies, many researchers integrated multiple models in the aforementioned domains to achieve convincing results that are applicable in real-world scenarios. Utilizing hybrid techniques, CNN with LSTM has achieved state-of-the-art results for various domains, such as convincing results for emotion recognition [13], speech processing [14], activity recognition [15] and also in the medical domain, where it shows superior performance in detecting arrhythmias [16]. Similar hybrid models are used in the energy forecasting domain to achieve state-of-the-art results.

Several techniques have been developed for energy consumption prediction, including ARIMA [17], SVM and SVR [18], time series [8], neuro fuzzy and linear regression (LR) models [19] and artificial neural networks [20]. These prediction models are grouped into four major groups: statistical, machine learning (ML), deep learning and hybrid models. Energy forecasting related studies are grouped based on this categorization and their descriptions along with the dataset used and strategy followed is given in Table 1.

Among the statistical-based models, Fumo and Biswas [21] used a linear regression model for residential energy prediction and observed time resolution effects on the model’s performance. Daily energy consumption prediction is proposed in Reference [22] by using multiple-linear regression with genetic programming. They integrated five variables through genetic programming and then fed them into their proposed prediction model. The performance of this model is increased by removing unnecessary variables, but independent variables correlation leads to the problem of multicollinearity and it is also challenging to get explanatory variables via linear regression models. Therefore, such models are not recommended for electricity prediction.

In the machine learning approaches category, SVR was used to forecast electricity consumption in buildings [23] and improved the performance of the model by adding temperature variables. Another approach based on random forest was developed in Reference [24], in which the authors predicted the following week’s energy by using human dynamics. In the machine learning approach, if the model does not have many features, then it generates complex decision boundaries. However, these models drain into an overfitting problem if the data is increased or the correlation between variables is complicated. If a model is overfitted, it greatly affects the prediction accuracy and hence is not recommended for use in residential or commercial buildings energy forecasting.

Deep learning models are widely used for electricity prediction, in which Reference [25] used a sequence-to-sequence model for electricity consumption prediction in buildings and achieved the highest possible performance. The authors of Reference [1] used stacked AE and reduced noise disturbance and randomness from the electricity consumption data via deep features. These models extracted important features in cases where they had complex attributes and a lot of redundant data. However, modeling the spatial and temporal features of electricity consumption data is difficult for deep learning models.

Among these approaches, some recent studies show combinations of models for electricity consumption prediction. The authors of Reference [26] integrated CNN with the LSTM model for electricity prediction, where the CNN layers were used to extract spatial features and LSTM was utilized for modeling temporal information. The combination of CNN with Bi-directional LSTM was presented in Reference [27] where the CNN layers were used to extract important information and the Bi-directional LSTM used these features in both the forward and backward direction to make a final prediction. These models achieved the best results but still the error rate was too high for them to be implemented for accurate electricity consumption prediction in real-world scenarios.

**Table 1:** The four types of prediction models for energy consumption.

Category	Paper	Learning Strategy	Dataset	Description
Statistical models	[21]	LR	Electricity consumption	Analysis of electricity prediction using LR according to time resolution.
	[22]	Multiple regression (MR)		Develops two models: ML and genetic algorithm (GA), where GA is used to select critical information from the dataset followed by optimal prediction via the ML model.
	[28]	MR		Uses backward elimination and a multicollinearity process for suitable variable selection and uses a MR model for medium-term electricity prediction.
Machine learning-based models	[23]	SVR	Electricity load	Adds a temperature variable to improve the performance of SVR for electricity prediction.
	[24]	Random forest regressor	Electricity consumption	Avoids overfitting by using an ensembled method and transforms the data from time to frequency domain to solve the input data computational complexity.
DL-based models	[25]	Seq2seq	Electricity load	Collects data from real smart meters and develops a sequence-to-sequence-based prediction model for short-term electricity prediction in buildings.
	[1]	Stacked AE (SAE)	Electricity consumption	Combines SAE with an extreme learning machine (ELM), where SAE is used to extract features and ELM is used as a prediction model.
	[29]	DRNN based on pooling	Electricity load	Uses pooling based DRNN, addresses the overfitting problem in a naïve deep learning network and tests the method in a real environment on smart meters in Ireland.
	[30]	Seq2seq	Electricity consumption	Uses a sequence-to-sequence model based on modified LSTM.
Hybrid models	[26]	CNN-LSTM		CNNs are used to extract spatial features and LSTM is used for modeling temporal features.
	[27]	CNN-bidirectional LSM		CNNs are used to extract spatial features and bidirectional LSTM is used for these features for final prediction.

We proposed a hybrid model of CNN LSTM-AEs’ synergy for electricity prediction in residential and commercial buildings. CNN layers are used to extract spatial features and their output is fed into LSTM-AE, followed by a dense (fully connected) layer for final prediction. Finally, the time resolution is changed to observe if further improvement can be made using the CNN with a LSTM-AE model. For the first time, a hybrid model of CNN and LSTM-AE is developed and tested to predict residential and commercial power consumption. The following are the main contributions of this research work:

- The input dataset is passed through a preprocessing step where redundant, outlier or missing values are removed, and the data are normalized to achieve satisfactory prediction results.
- A novel hybrid model is developed in this work for accurate future energy prediction. The proposed model integrates CNN with LSTM_AE in which the CNN layers are used to extract spatial features from input data and then LSTM-AE are used to model these features.
- The experimental results demonstrate that the proposed CNN with LSTM-AE model has the best performance compared to other models. The evaluation metrics record the smallest value for MSE, MAE, RMSE and MAPE for energy consumption prediction.

## 2. Proposed Framework

**Figure 1:**
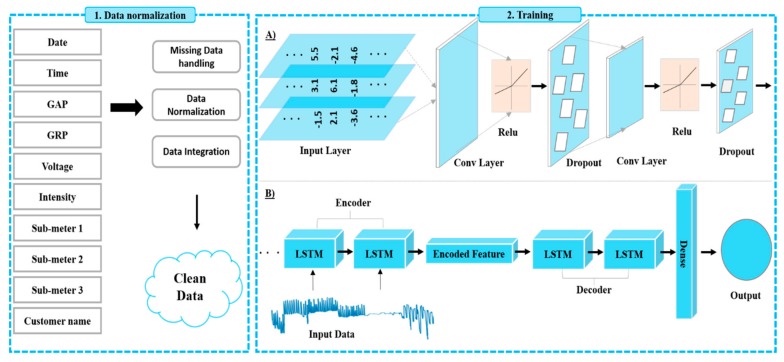
Proposed framework for electricity consumption prediction.

Prediction of electrical power consumption in residential and commercial buildings is very important to provide better energy management services. Due to the impact of unpredictability or the noisy arrangement of data, accurate electricity consumption prediction is a challenging task. For these reasons, the forecasting model sometimes generates incorrect prediction results. Moreover, several methods have been developed based on traditional networks with high error rates. The traditional methods have the problems of needing to learn from scratch, overfitting or short-term memory challenges if the data increase or the correlation between variables is complicated. These issues can be easily solved using sequential learning models, through modeling the spatial and temporal features for electricity consumption is also challenging. Therefore, in this paper, we developed a CNN with LSTM-AE model and a data preprocessing step to efficiently predict electricity consumption in residential and commercial buildings. The overall architecture of the proposed framework for electricity consumption is shown in Figure 1. Further, each section of the proposed framework for electricity consumption is discussed in the next sections.

### 2.1. Data Preprocessing

This section offers detailed analysis about the collection and refinement of data. The data is collected from smart meters which are installed at the edge of the electricity network and connect all appliances to a main board. Normally, the data are gathered annually or monthly, which generates noise and abnormalities in the data due to measurement or human error, meter problems and climate change, if the meters are installed for a long time. Before training, the data need to be refined and normalized for good results.

**Figure 2:**
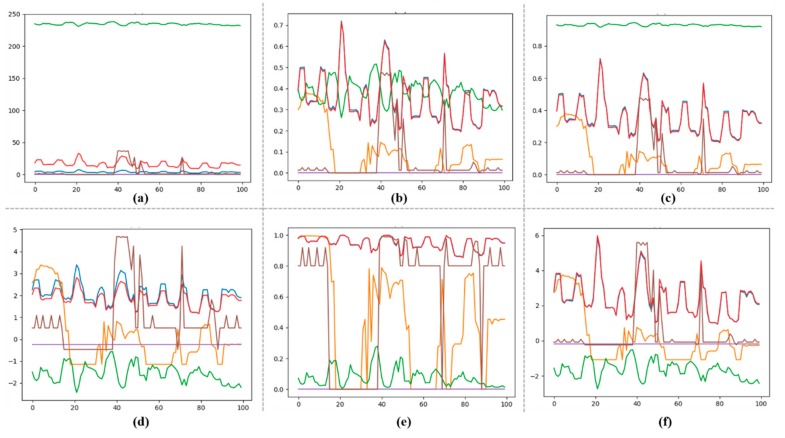
Data normalization techniques, where (**a**) original data in the dataset, (**b**) the range of data after applying Min-Max scalar, (**c**) the range of data after applying Max-Abs scalar, (**d**) the range of values after applying power transform, (**e**) the data plot after quantile transformation, and (**f**) the range of data after applying standard transformation.

The tested datasets include null, redundant and outlier values. Similarly, samples from the datasets are not all in the same range and need to be normalized before training for accurate prediction. Null, redundant and outlier values are extracted from the datasets and are discussed in this section. Also, different normalization techniques were applied to get the odd range values within a specified limit. These techniques include Min-Max scalar, standard transform, Max-Abs scalar, quantile and power transform, as shown in Figure 2. After detailed analysis of each technique, finally, we selected standard transform for data normalization because it centers and scales each feature independently.

The range of each feature is different in the original dataset, as shown in Figure 2a where the ranges of features are between 0–10, 0–50 and 200–250. After applying Max-Min normalization technique, the range of these features lies between 0 and 0.7, as visualized in Figure 2b. Similarly, after processing data with Max-Abs, the ranges are normalized between 0 to 0.8, as shown in Figure 2c. After normalizing data with quantile transformation, the features range is achieved between 0 to 1, as visualized in Figure 2e. However, we needed to transform the input data in a way such that the negative values also exist in the features to achieve good results. The range of power transformation is between −2 to 5 as visualized in Figure 2d, and standard transform is −2 to 6 as given in Figure 2f. However, the computational complexity of power law transformation is higher than standard transformation. Also, standard transform processes each feature independently. Due to these reasons, finally, we selected standard transform for data normalization.

### 2.2. ANN

**Figure 3:**
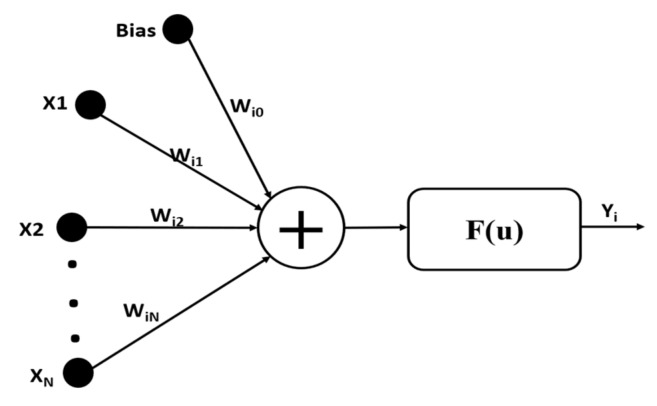
The simple neuron operation in ANN, where “X” represents the input data, “W” represents the weights, ”F” is the activation function and “Yi” is the output.

ANN is a type of strong mathematical modeling tool inspired by the human nervous system. An early ANN model is MLP [31] which includes input, hidden and output layers. Each neuron relates to the next and previous layer neurons, which are similar in MLP with several input and output links. The value retrieved from the previous layer is summed up with some weight for each neuron individually, and a bias term. Finally, activation function “f” is used to transform the sum, which may be different for each neuron, as shown in Figure 3.

### 2.3. CNN

**Figure 4:**
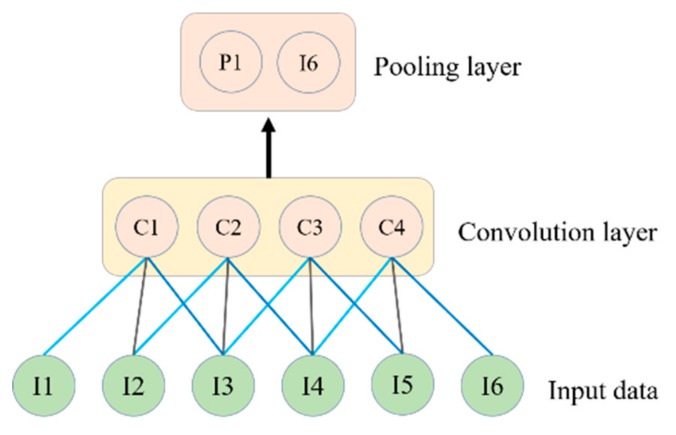
The operation of convolution layers and pooling layers over input data.

CNN was specially developed for grid topology data processing [32]. For example, visual data, i.e., images and videos, are viewed as a two-dimensional grid and time series data are viewed as one-dimensional data. The CNN [33,34,35] uses a weight sharing concept that provides high accuracy in nonlinear problems, such as energy consumption prediction. Convolution-pooling layers of one dimension are shown in Figure 4. When the convolution is applied to the input data, I1, I2, I3, I4, I5 and I6 are converted to a features map C1, C2, C3, C4. Next, a pooling layer is applied to sample the feature-maps of the convolution layer. The pooling layer procedure is important for extracting high-level convolution features; after applying the pooling layer, the dimension of the features map is reduced to 2.

### 2.4. LSTM

**Figure 5:**
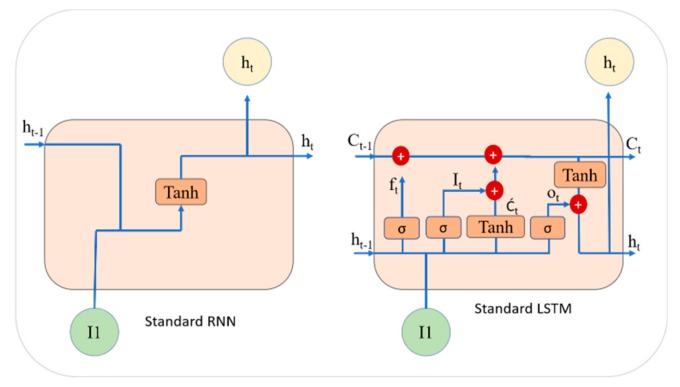
Standard architecture of RNN and LSTM.

The recurrent neural network (RNN) is another popular deep learning architecture, where connections between units form a directed graph along with the sequence information from the input, as depicted in Figure 5. The RNN processes a sequence of input data by using their internal state and turns into a vanishing gradient problem, which has a major negative effect on the model accuracy. An enhanced version of RNN is LSTM [36], which overcomes the vanishing gradient problem via the concept of gates (input, forget, and output) and memory cells. The LSTM operation is illustrated by the following equations and its structure is shown in Figure 5.

(1) $$f_{t}=\;\Phi \;\left({Ŵ}_{f}\;\cdot \;\left[h_{t-1},\;x_{t}\right]\;+\;B_{f}\right)$$

(2) $$i_{t}=\;\Phi \;\left({Ŵ}_{i}\;\cdot \;\left[h_{t-1},\;x_{t}\right]\;+\;B_{i}\right)$$

(3) $${Ċ}_{t}=\;\tanh\;\left({Ŵ}_{C}\;\cdot \;\left[h_{t-1},\;x_{t}\right]\;+\;B_{C}\right)$$

(4) $$C_{t}=\;f_{t}\times C_{t-1}+\;i_{t}\times {Ċ}_{t}\;$$

(5) $$o_{t}=\;\Phi \left({Ŵ}_{o}\;\cdot \;\left[h_{t-1},\;x_{t}\right]\;+\;B_{o}\right)$$

(6) $$h_{t}=\;o_{t}\times \tanh(\Phi \left(C_{t}\right).$$

In Equation (1), the network input is *x_t_*, *h_t_* is the output of the hidden layer, *Φ* represents the sigmoid function, the cell state is *C_t_* and the state candidate values are represented through *Ċ_t_*. Ŵ*_i_*, Ŵ*_o_*, Ŵ*_f_* and Ŵ*_C_* are the weights for the input, output, forget gate and memory cells, while *B_i_*, *B_o_*, *B_f_* and *B_C_* represent the bias for the input, output, forget gate and cell, respectively. The input gate decides whether input data will be reserved or not, the forget gate verifies if data will be lost or not, the cell records the processing state and the output is delivered through the output gate. This architecture is specially designed to address the vanishing gradient problem in RNNs.

### 2.5. LSTM-AE

**Figure 6:**
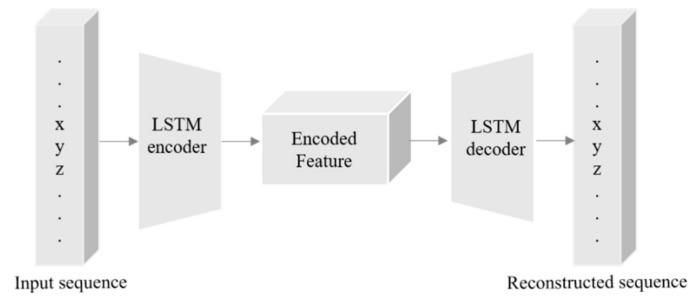
The internal structure of LSTM-AE where the first LSTM layer used as an encoder and the second is a decoder.

Autoencoders (AE) are generally used in representation learning to understand unsupervised inputs in a feature vector. The conventional method utilizing an LSTM-AE is illustrated in Figure 6. We employed sequence-to-sequence AE for a time-series sequence dataset. The optimal goal is to predict the short-term electricity consumption of residential and commercial buildings. AE consists of an encoder and a decoder, where the input sequence is first encoded and then decoded. Let xt be the input features and F the feature space. The encoder function applied is: φ: xt →F that learns important features and encodes the features vector F. In the decoder, Ɖ = F → X, which intends to reconstruct the input by utilizing internal representations [37]

We employed LSTM cells for the execution of the encoder and decoder, which are capable of learning from temporal dependencies from one sequence and another. Formally, for input samples sequence *X*(*N*), the *AE* function is applied *ΦAE*: φ Ɖ, which outputs samples *x*(*N*).

(7) $$\Phi_{AE}\left(X\left(N\right)\right)=\;x\left(N\right)$$

### 2.6. Training

In our proposed framework, the refined input data is passed to the training step. The training step includes two sub-sections where “A” demonstrates the CNN architecture and “B” shows the LSTM-AE architecture. The proposed hybrid model combines CNN with LSTM-AE to predict hourly and daily electricity consumption for residential and commercial buildings. The CNN layers include an input layer, hidden layers and an output layer, which extract features for LSTM-AE. The hidden layers include convolution, dropout, pooling and ReLU layers. Two convolution layers with the RELU activation function and dropout layer after each convolution are employed. Initially, the CNN extracts feature from the refined input data, then the output CNN features are fed into the LSTM encoder, which encodes the input sequences of four time-steps. The repeated vector layer replicates these encoded sequences twice from the model. These encoded sequences are inputted into another LSTM for decoding and finally a dense layer is used to produce the output prediction for the input sequence. The LSTM has problems modeling spatial features, so in this work we used CNN to extract spatial features and then fed them to the LSTM. Normally, the LSTM fails to learn temporal dependencies from one sequence to another, so in this work we developed a hybrid network to tackle these issues and developed a reliable solution for accurate electricity prediction. In this architecture, we used two 1D-convolutional layers, where two dropout layers are inserted after each convolutional layer, two encoder LSTM layers, one repeated vector layer, two decoder LSTM layers and finally one fully connected layer. As a result, the total number of layers are 10 in the proposed architecture and the model size is 445 KB with 33,811 parameters. The filter size for first convolution layer is 8 while for the second layer it is 16 and the kernel size is one for both convolution layers.

The proposed method works better than other state-of-the-art models because we integrated multiple architectures to develop a hybrid model (CNN-LSTM-AE), where CNN is used to extract spatial features from the input dataset and then feed these features to LSTM-AE. The simple LSTM model works well but is unable to learn temporal dependency between sequences, while LSTM-AE is capable of learning from temporal dependencies from one sequence and another. This is experimentally proven and the results are discussed in the Section 3. Therefore, we claim that our model works well and show convincing results when compared to other models.

## 3. Results

This section provides detailed discussion about the experimental setup, datasets, evaluation metrics, evaluation of the UCI dataset, evaluation of the Korean commercial building dataset and finally a comparative analysis of the proposed hybrid network with other baseline models.

### 3.1. Experimental Setup

We evaluated and validated the efficiency of the proposed hybrid CNN with LSTM-AE model using residential and commercial buildings datasets. We trained our hybrid model on TITAN X (Pascal)/PCLe/SSE2 GPU with an Intel Core i5-6600 processor, with 64 GB memory over the Ubuntu 16.4 LTS operating system. This model was implemented in Python (V3.5) in Keras (V2.2.4) with a TensorFlow (V1.12) backend and employed Adam as the optimizer. Several experiments were conducted to find the optimal selection of the hyper perimeter of each model. After extensive experiments, finally we decided to train the model over 50 epochs with 1000 as the batch size and a 0.2 validation split.

### 3.2. Datasets

In this paper, we used two datasets: the household electric power consumption dataset available on the UCI machine learning repository [38] and our own commercial data. A number of time-series variables were used in the proposed architecture to predict the global active power consumption. The UCI dataset contains actual power consumption data, with one-minute resolution, collected from a single residential building in France between 2006 and 2010. A total of 2,075,269 records are present in the dataset, with 25,979 missing values that are handled in the preprocessing step of the proposed framework. The dataset is then grouped into hourly and daily resolution to predict the electricity consumption for the short term. Table 2 shows the electricity consumption variables of the UCI dataset, which include date, time, global active power, global reactive power, voltage, intensity, submetering_1, submetering_2 and submetering_3 variables. The time variable includes months, days, years, hours and minutes. The submetering shows the electricity consumption in the home, where submetering_1‒3 corresponds to the kitchen, laundry room and living room, respectively.

Our new dataset is similar to the UCI dataset but with some differences which are mentioned below:

- The UCI dataset was derived from residential buildings while the proposed dataset was generated in commercial buildings.
- The UCI dataset has three consumption sensors: submeters 1, 2 and 3, while our dataset includes only one electricity consumption sensor.
- The UCI dataset includes 1-minute resolution, while the proposed dataset has 15-minute resolution.

### 3.3. Evaluation Metrics

**Figure 7:**
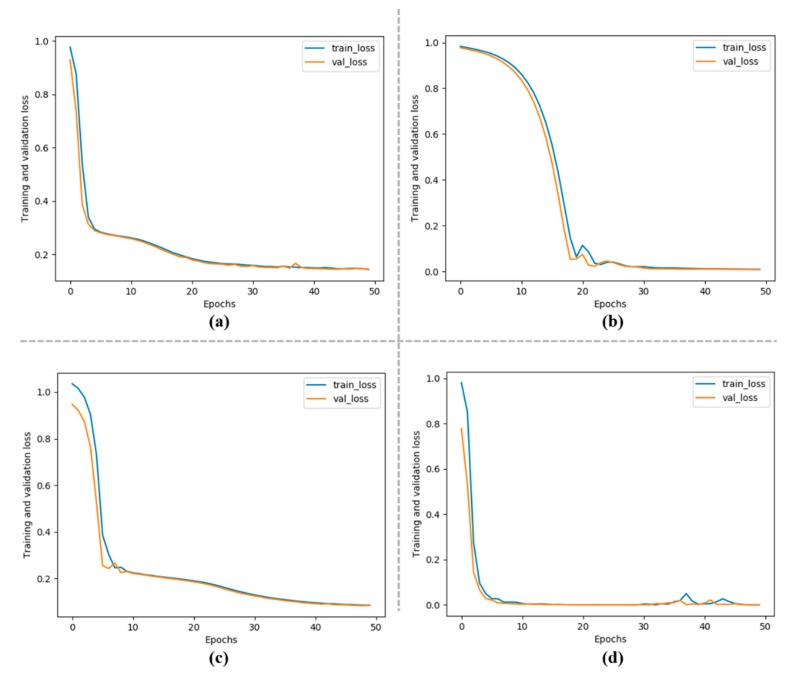
Training and validation loss during training.

The proposed method is evaluated on four standard metrics: MSE, MAE, RMSE and MAPE. The mathematical formulas of these metrics are given in Equations (8)–(11). RMSE is the percentage of difference between predicted and testing variables, MAE represents the percentage of difference between the predicted variables, MSE represents the average square value between the testing and predicted variables, while the last metric MAPE expresses the prediction accuracy in percentage. The training and validation loses for both UCI and Korean commercial building dataset are shown in Figure 7, where “A”, “B”, “C” and “D” represent the loses for residential building hourly data, residential building daily data, Korean commercial building hourly data and Korean commercial building daily data, respectively.

There are a total of 960,000 records in our dataset, with null and redundant values that are removed in the preprocessing step. Next, we normalized the input data to train the proposed model efficiently. For training purposes, 75% of the data are used from each dataset, while the remaining 25% are used for testing. This means that the first three years data of the UCI dataset are used for training, while the last year’s data are used for testing. Furthermore, we performed several experiments on different deep models for comparison, such as CNN, LSTM, LSTM-AE and the CNN with LSTM-AE models.

(8) $$MSE=\;\frac{1}{n}\sum_{1}^{n}\left(y-\hat{y}\right)2$$

(9) $$MAE=\;\frac{1}{n}\sum_{1}^{n}\left|y-\hat{y}\right|$$

(10) $$RMSE=\;\sqrt{\frac{1}{n}\sum_{1}^{n}(y-\hat{y})}$$

(11) $$MAPE=\;\frac{100\%}{n}\sum_{t-1}^{n}\left|\frac{A_{t}-F_{t}}{A_{t}}\right|$$

### 3.4. Performance Evaluation over UCI Dataset

**Figure 8:**
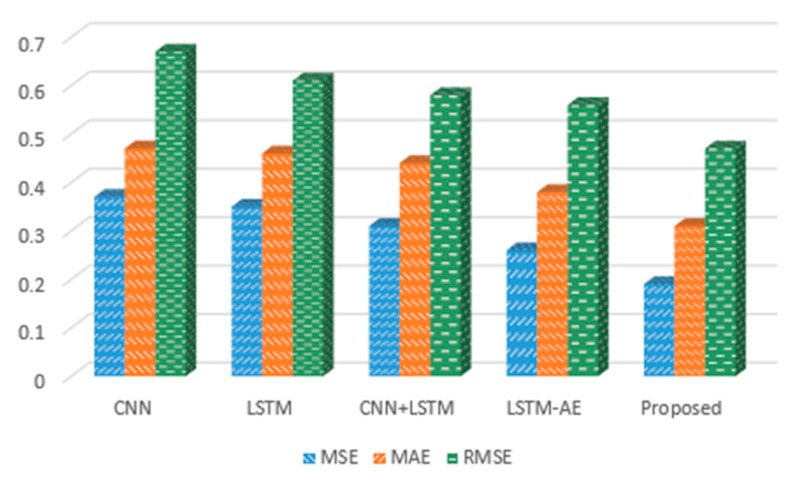
The MSE, MAE and RMSE error rates of different deep learning models for hourly electricity prediction.

To validate the robustness of the proposed hybrid model, we performed experiments on several deep learning models with variable sets of resolutions. The results achieved for each model over hourly data are shown in Figure 8. First, we used CNN to check the performance of the model, and obtained values of 0.37, 0.47 and 0.67 for MSE, MAE and RMSE, respectively. On the other hand, when using LSTM, we observed 0.35, 0.45 and 0.61 for MAE, MSE and RMSE, correspondingly. Moreover, with the combined CNN-LSTM we obtained 0.31, 0.44, and 0.58 for MSE, MAE, RMSE, and with the LSTM-AE model values of 0.26, 0.38 and 0.56 for MSE, MAE, and RMSE, respectively. Inspired by the results of LSTM-AE, we combined CNN with LSTM-AE and recorded the smallest values: 0.19, 0.31 and 0.47 for MSE, MAE and RMSE, respectively.

**Figure 9:**
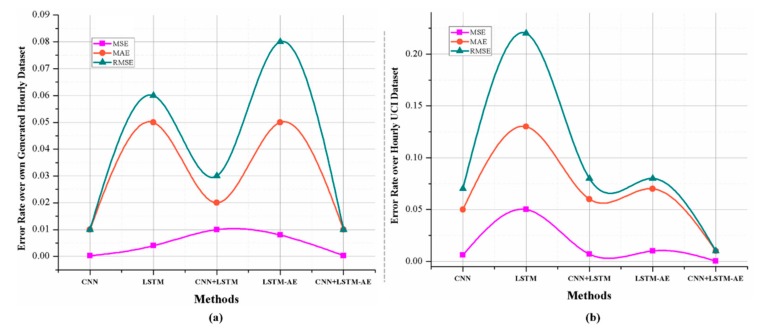
The detailed results of different deep learning-based models for one day resolution data where (**a**) demonstrates MSE, MAE and RMSE for the Korean commercial building dataset and (**b**) shows these error rates over UCI dataset.

Next, the performance of the aforementioned deep learning models for daily data was tested. For the MSE, MAE and RMSE evaluation metrics, our method performed best compared to the baseline models. In more detail, CNN achieved values of 0.006, 0.05 and 0.07 for MSE, MAE and RMSE, respectively, while LSTM reduced its error rate (compared with the hourly rate) to 0.05, 0.13 and 0.22 for MAE, MSE and RMSE. Furthermore, we combined the CNN with LSTM and achieved 0.007, 0.06, and 0.08 for MSE, MAE, and RMSE, whereas LSTM-AE showed values of 0.01, 0.07 and 0.11 for MSE, MAE, and RMSE, respectively. Finally, we tested the proposed CNN with LSTM-AE hybrid model and obtained the lowest values of all, at 0.0004, 0.01 and 0.02 for MSE, MAE and RMSE, respectively, as shown in Figure 9b. 

### 3.5. Performance Evaluation over Newly Generated Dataset

**Figure 10:**
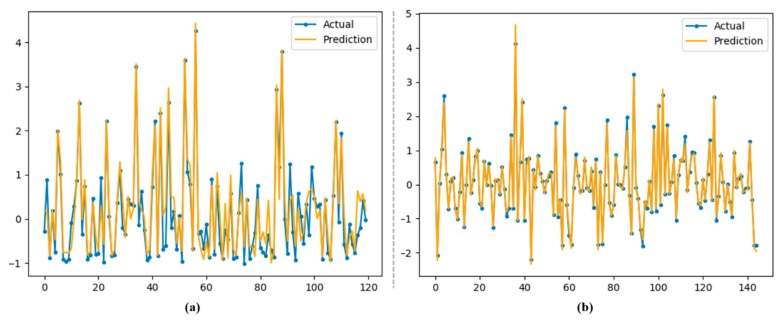
Visualization of performance of our proposed CNN with LSTM-AE over testing data for electricity prediction. (**a**) electricity consumption prediction for hourly data; (**b**) electricity prediction for daily data.

The aforementioned models were also tested on our newly generated dataset, and the proposed model recorded convincing values for the tested evaluation metrics. The dataset was tested on both hourly and daily data resolution, as shown in Figure 10 where (a) shows electricity consumption prediction for hourly data, while (b) indicates electricity prediction for daily data. The difference between actual and predicted values is very narrow, but better performance is evident for the proposed model, especially for daily data future load prediction. 

**Figure 11:**
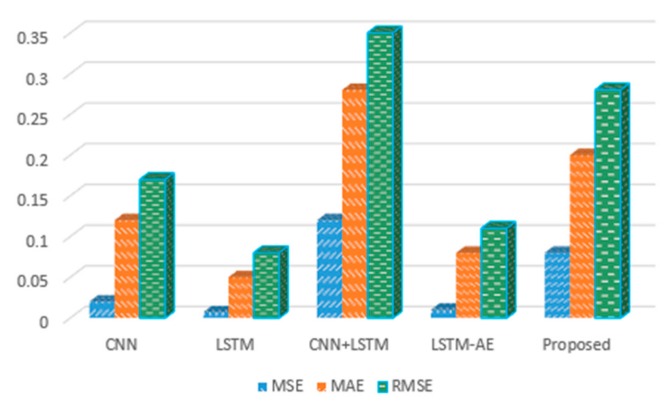
The results achieved by different deep learning-based models for daily resolution of data on our own dataset.

For hourly electricity prediction on the Korean commercial building dataset, the proposed model stands in third place, LSTM-AE is second and LSTM is first. For daily electricity prediction, the proposed model achieved the lowest error rates of 0.0003, 0.01 and 0.01 for MSE, MAE and RMSE, respectively. Figure 9a shows the prediction performance of the proposed hybrid model for hourly electricity consumption, while Figure 11 demonstrates the daily energy prediction error rate for each model.

### 3.6. Comparison with other Baseline Models

**Figure 12:**
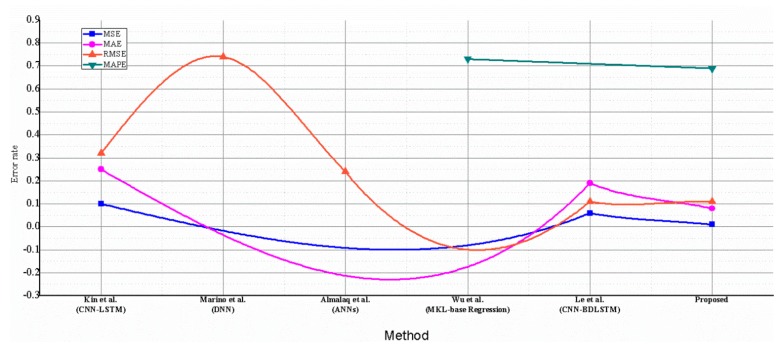
Comparative analysis of the proposed hybrid CNN with LSTM-AE model with the methods developed by Kim et al. [26], Marino et al. [30], Almalaq et al. [40], Wu et al. [41] and Le et al. [27]. In the figure, our model performance is compared with other state-of-the-art models in term of MSE, MAE, RMSE and MAPE. Our model attains the smallest values for each metric.

The performance of the proposed hybrid model was evaluated and compared with other competitive baseline models, which were similarly used for the same dataset. The results were compared for both hourly and daily data. For hourly prediction, the proposed method was compared with References [26,27,30,39] and achieved the smallest error rate among these models, as shown in Table 3. For daily prediction, the proposed model performance was compared with References [26,27,30,40,41] and achieved better results, as demonstrated in Figure 12. For instance, the proposed hybrid model recorded the smallest error rates of 0.19, 0.31 and 0.47 for the hourly dataset, and recorded 0.01, 0.08, 0.11 and 0.69 for the daily dataset.

## 4. Conclusions

In this article, we developed a novel framework for the prediction of electricity consumption in residential and commercial buildings, and evaluated it using two datasets including the UCI household electricity consumption prediction and Korean commercial building data. Initially, the input data are preprocessed to remove missing, redundant and outlier values. Next, we apply different normalization techniques for better representation of the input data, which yields an effective model. Further, we developed a novel hybrid CNN with LSTM-AE model. The proposed model has three modules for predicting electricity consumption: CNN, LSTM-AE and FC. Primarily, two CNN layers are used to extract information from several variables in the dataset, which are then fed to LSTM-AE, which converts the sequence into an encoded features vector and then decodes it through another LSTM. The encoded feature vector layer duplicates these encoded sequences and finally a dense layer is used to produce the output prediction. The experimental results of the proposed hybrid model outperform other state-of-the-art models for electricity consumption prediction, in terms of different performance metrics such as MSE, MAE, RMSE and MAPE.

## Figures and Tables

**Table 2:** Feature representation and detailed description of the residential dataset, namely the “individual household electricity consumption dataset”.

Variable	Description
Date	Presented in dd/mm/yyyy format.
Time	Time variable given in hours, minutes and seconds (hh:mm:ss)
Global active power	Minutely given average active and reactive power for individual house.
Global active power	
Voltage	One-minute average voltage
Intensity	Current intensity for every minute.
Submetering (1, 2, 3)	Active electricity related to kitchen, laundry room and living room of residential home, while only one submetering_1 sensor in commercial dataset is related to office electricity.

**Table 3:** The comparative analysis of the proposed method with other state-of-the-art Deep Learning and traditional techniques for hourly data resolution.

	Methods	MSE	MAE	RMSE	MAPE
Deep Learning Methods	Kim, T.-Y et al. [26]	0.35	0.33	0.59	-
	Kim, J, -Y et al. [39]	0.38	0.39	-	-
	Marino et al. [30]	-	-	0.74	-
	Le et al. [27]	0.29	0.39	0.54	-
Traditional Machine Learning models	ARMA [42]	-	-	0.30	-
	SVM [43]	-	1.12	1.25	-
	Linear Regression [41]	-	-	-	1.03
	SVR [41]	-	-	-	1.29
	Gaussian Process [41]	-	-	-	0.82
	**Proposed**	**0.19**	**0.31**	**0.47**	**0.76**
